# Traditional Tar Production from the Anatolian Black Pine [*Pinus nigra* Arn. subsp. *pallasiana* (Lamb.) Holmboe var. *pallasiana*] and its usages in Afyonkarahisar, Central Western Turkey

**DOI:** 10.1186/1746-4269-10-29

**Published:** 2014-03-27

**Authors:** Süleyman Arı, Mustafa Kargıoğlu, Mehmet Temel, Muhsin Konuk

**Affiliations:** 1 Department of Biology, Faculty of Science and Literatures, Afyon Kocatepe University, 03200 Afyonkarahisar, Turkey; 2 Department of Molecular Biology and Genetics, Faculty of Engineering and Natural Sciences, üsküdar University, 34662 Istanbul, Turkey

**Keywords:** Tar, Black pine, *Pinus nigra* subsp. *pallasiana*, Afyonkarahisar, Turkey

## Abstract

**Background:**

Tar is one example of a plant product used in folk medicine and it is obtained from *Pinus nigra* Arn. subsp. *pallasiana* (Lamb.) Holmboe, which is very common in the West Anatolian Region. Old trees that are good for kindling and have thick trucks are preferred to obtain tar. Tar is used not only as traditional medicine but also for protection against both endoparasites and ectoparasites. The objective of this study was to record the traditional method of obtaining tar and its usages in Afyonkarahisar which is located in the Western Anatolian Region of Turkey.

**Methods:**

In order to record the traditional methods of obtaining tar, we visited the villages of Doğlat, Kürtyurdu and Çatağıl in Afyonkarahisar (Turkey) June-July, 2012. Ethnobotanical data about the method of collection and traditional usages of tar were obtained through informal interviews with 26 participants (16 men and 10 women). Data concerning the method of tar collection and its traditional usages were recorded and photographed.

**Results:**

The traditional method for obtaining tar from *Pinus nigra* subsp. *pallasiana* by local people was recorded and the local usages (curing ear pain in children, osteomyelitis, wounds, ulcers, eczema, acne, alopecia, fungus, foot-and-mouth disease in animals, mouth sores in sheep and goats, protection against endo- and ectoparasites, repellent for snakes, mice, flies (*Tabanus bovinus*) and ticks*,* and the prevention of water leakage from roofs) of tar are described.

**Conclusion:**

In this study, the traditional method for obtaining tar and the traditional usages of tar are explained. Documentation of the method of obtaining tar and its traditional usages may contribute to scientific research on the benefits and usages of tar in medicine, veterinary medicine, as well as other fields.

## Background

Turkey is one of the richest countries in the world in terms of plant diversity. There are more than 10,000 plant species within its borders, and 30% of these are endemic [1,2]. Many plant species have been widely used as traditional medicine, tea, spice, food, firewood, dye, furniture, agricultural tools, construction materials and indoor plants by Turkish people [3-13]. The term traditional medicine describes the usage of natural resources in order to prevent, treat, and heal human diseases and ailments [14]. Secondary products of forest trees have been used both as a natural medicine and surface coating material. Some woody trees (*Juniperus* sp., *Pinus* sp. *Picea* sp., *Cedrus* sp., *Betula s*p. and *Fagus* sp.) have been used for tar production since ancient times. Tar production from *Pinus sylvestris* L. (Scots pine) and *Cedrus libani* A. Rich. (Lebanon or Taurus cedar) have historical importance and a wide range of applications [6,15-17].

Five *Pinus* species (*Pinus brutia* Ten., *Pinus halepensis* Mill., *Pinus nigra* Arn., *Pinus pinea* L. and *Pinus sylvestris* L.) are found in Turkey. There are five taxa of *Pinus nigra* Arn. (Black pine): subsp. *pallasiana* (Lamb.) Holmboe var. *pallasiana* (Anatolian black pine)*,* var. *fastigiata* Businsky, var. *seneriana* (Saatçioğlu) Yaltırık, var. *columnaris-pendula* Boydak, var. *yaltirikiana* Alptekin. The black pines are found in almost 4.2 million ha (hectare) in the Black Sea, Marmara, Aegean, Taurus Mountain and Central Anatolia regions. Pines are the most valuable trees in the world due to their many different usages, especially as timber and wood. In Turkey, pine wood has been widely used for furniture, window frames, paneling, floors, roofing, cellars and paper and a variety of other products such as resin (an important source of turpentine), tar and edible seeds produced from *P. pinea*. The various parts of *Pinus* species have ethnobotanical usages (derived from resin, cones, tar and pine honey) and ethnomedicinal usages (as treatments for skin conditions, asthma, wounds, bronchitis, the common cold and cough) [18]. The turpentine of *Pinus nigra* subsp. *pallasiana* has strong antioxidant and analgesic effects [19].

In the past, tar was obtained using stable ovens. The quality of the tar is influenced by the amount of resin in the wood, the age of the tree and the type of method used to extract the tar. In the Ottoman Period, tar production was 2830 quintal per year. Half of the tar that is extracted is black tar and the other half is yellow. Yellow tar is mainly obtained from *Juniperus* spp. and *Cedrus libani* while black tar is obtained from *Pinus nigra* subsp. *pallasiana* and *Pinus brutia*[15,20].

Today, tar is often obtained in modern laboratories. The quality and usefulness of the tar obtained by laboratories and traditional methods differs. Tar obtained from *Cedrus libani* in southern Turkey is used to protect wooden structures against insects and fungi, as anti-parasitic and anti-bacterial agents, and is used to aid in wound healing and cure various external and internal diseases in humans and domestic animals. Tar is called ‘katran’ in the Turkish language [16]. It is generally obtained from *Pinus nigra* subsp. *pallasiana* in the Afyonkarahisar province, which has about 2,500 plant taxa [21].

The objectives of this study are to describe the traditional method for obtaining tar from *Pinus nigra* subsp. *pallasiana* and the traditional uses of pine tar (katran) by local people.

## Methods

### Study area and features the of villagers

**Figure 1:**
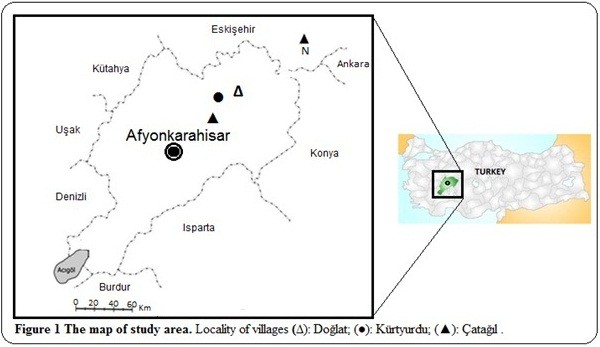
**The map of study area.** Locality of villages (∆): Doğlat; (●): Kürtyurdu; (▲): Çatağıl.

The province of Afyonkarahisar is located in the central Western part of the Aegean region of Anatolia and phyto-geographically located on the transition zone between the Irano-Turanian and Mediterranean regions. The area also has many Euro-Siberian (Euxine) phyto-geographic elements. The villages of Doğlat, Kürtyurdu and Çatağıl are located in northeastern part of Afyonkarahisar (Figure 1) and share a similar cultural heritage with the other villages of Afyonkarahisar. The altitude of the study area is almost 1050 m. The region has a continental climate. The average annual temperature is 10.7°C. The average temperature in July is 20°C. The temperature in January is 4.8°C. The annual amount of rainfall is 388 mm [21,22].

The Beydağları, Eğerli, Asarkale, and Köroğlu Mountains are near villages and an important reservoir of black pine trees used for obtaining tar.

The income of the villagers in the study area is derived mainly from livestock and agriculture. The populations of Doğlat, Kürtyurdu and Çatağıl are 242, 232, and 135, respectively. The highest level of education in the majority of the villagers is primary school. Until recent times, the Turkish government only required that citizens complete education through primary school. The people who were interviewed in this study were recommended by local people in the tea houses in the three villages, which serve the same role as a local pub in many villages. Every village has at least one tea house. Twenty-six people were interviewed, and the majority of these people were older than 60. They all had practical experience in obtaining tar and its traditional usages. Sixteen of the participants were men and ten were women. We went to the participants’ houses at a pre-arranged time to gather data about the method of tar collection and its traditional usages through informal interviews. Photographs were taken during the interviews.

### Extraction process

**Figure 2:**
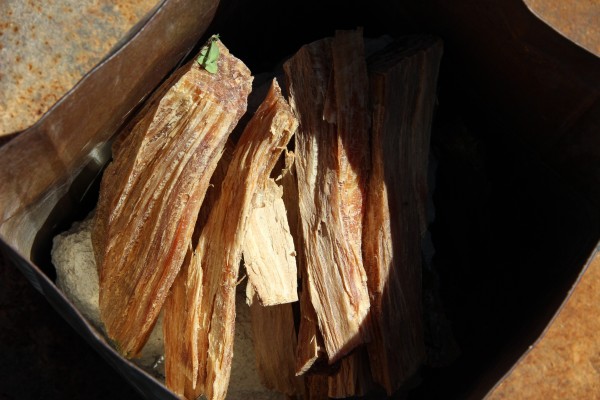
The kindling (Çıra) used by the local people to produce tar.

In order to obtain tar using the traditional method, a hole is dug that is 60 cm in diameter and 30–35 cm deep. The hole is covered with stones. This stone-covered hole is turned into an oven by covering the stones with a mixture that includes scat, hay and sand. Two holes are dug into the oven and are connected to each other. A big basin is put under one of the holes. Also, a trivet is put into the basin. The trivet forms a space between the basin and the bowl, which contains the kindling. The bowl on the trivet that contains the kindling is made of tin and is about 60–70 cm in height and 30–40 cm in width. Holes that are big enough for tar to run through are put into the bottom of the bowl before the kindling is placed into it. Kindling that is about 30 cm long is packed into the bowl and no space is left in between the pieces of kindling. If space is left between the kindling, the air will hasten the formation of the tar which decreases the quality of the tar. The top of the bowl with the kindling is covered with mud which further prevents the introduction of excess air. Using this method, the kindling burns slowly. When the kindling burns slowly, higher quality tar is produced. The burnt kindling falls from the bowl through into holes beneath the bowl while slowly burning at 300°C. A connection between the second and first holes is created via metal pipes. Tar is transferred from the bowl over the 1^st^ hole to a bowl over the second hole. The pipes are connected in a manner than prevents contact with air. It takes 7–8 hours to obtain tar (Figure 2).

## Results and discussion

Tar was obtained successfully from black pine (*Pinus nigra* subsp. *pallasiana*) by the traditional methods utilized by people in three rural Turkish villages. Local people have used tar to cure diseases and care for various animals. Traditional medicine is often preferred by the villagers because they live in a mountainous region that is far from hospitals. For example, it is common to apply tar to the ear of a baby that is suffering from otalgia (Table 1).

**Table 1:** The usages of tar in the study area and from literatures in Turkey

**Kind of usages**	Usages in the study area/application type*	Usages in Turkey/application type*/[Literature]
Ethnomedicinal (for human and animal) usages	- To cure Osteomyelitis/Ext.	-Antiseptic in both the respiratory and urinary tracts, dermatological diseases/Int., Ext./ [6]
	-Ear infection (otalgia)/Ext.	
	-Skin wounds/Ext.	-Treatments for skin conditions, asthma, wounds, bronchitis, the common cold and cough/Ext., Int., Inf./ [18]
	-Ulcer/Int.	
	-Eczema/Ext.	
	-Acne/Ext.	-Eczema, acne, alopecia, fungus, mange and psoriasis/Ext./ [24]
	-Alopecia/Ext.	
	-Fungus/Ext.	-Common cold, diaphoretic, skin softener, pain reliever and muscle relaxant/Ext./ [26]
	-To treat skin diseases such as mange on goats and sheep/Ext.	
	-Foot-and-mouth disease of sheep and goats/Ext., Int.	-Antiseptic on dermatologic lesions and as a topical antifungal on the skin. Respiratory tract and urinary tract diseases/Ext., Int./ [27]
	-Viral infections/Int., Ext.	-Oral antiseptic and for covering wounds/Int., Ext./ [28]
	-To protect the digestive system from endoparasites/Int.	
	-To cure wounds inflicted by wolves/Ext.	-To treat stomachaches in children and cracked hands and feet during the winter and for calluses/Int., Ext./ [29]
	-Rolling into the feet to kill the bacteria *Mycoplasma agalactiae* causing a reduction in goat’s milk/Ext.	-To treat ulcers, ectodermal parasites, wounds, cuts, bruises, asthma and upper respiratory disease/Int., Ext./ [16,31]
	-To cure wounds, scars and purple spots created by *Tabanus bovinus*/Ext.	-To kill the bacteria *Mycoplasma agalactiae* causes a reduction in goat’s milk/Ext./ [26,32]
		-To cure for fluke disease in sheep/Int./ [33]
		-To kill the *Varroa destructor* parasite/Inf./ [34]
Technical	-To prevent water leaks from wooden roofs/Ext.	-Coating on wooden products for houses and stables/Ext./ [16]
		-On boats, ships, and ropes as waterproofing and protection against fungi and aquatic animals/Ext./ [20]
Repellent or insecticide	-Acts as a snake and rodent repellant/Inf.	-To repell ticks, fleas, mosquitoes, and horseflies, snake, mice, spides, and scorpion/Ext., Inf./ [16]

*Ext.: External, Int.: Internal, Inf.: Infusion.

**Figure 3:**
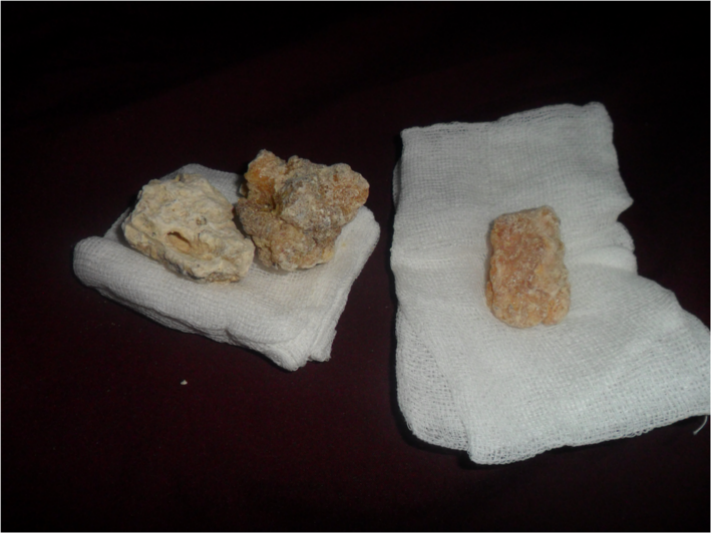
Applying the mixture of black pine tar and gum on the waist.

**Figure 4:**
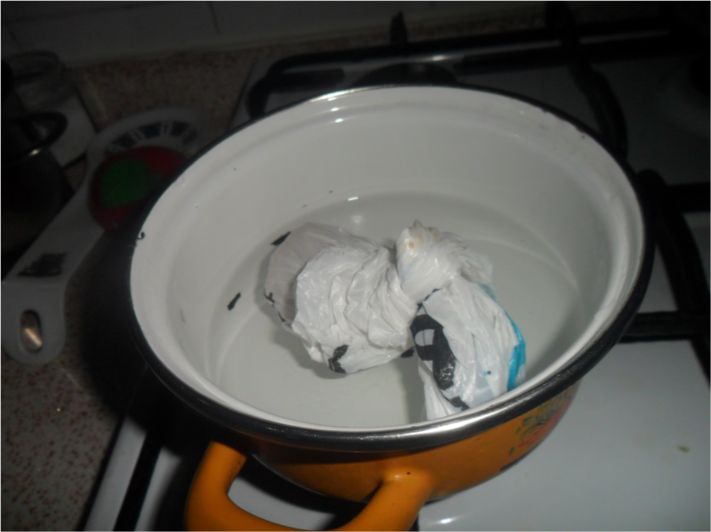
Applied form of tar on the waist.

**Figure 5:**
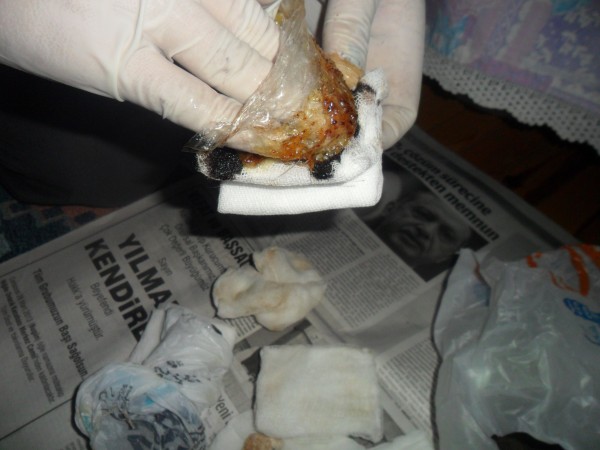
Black pine gum.

**Figure 6:**
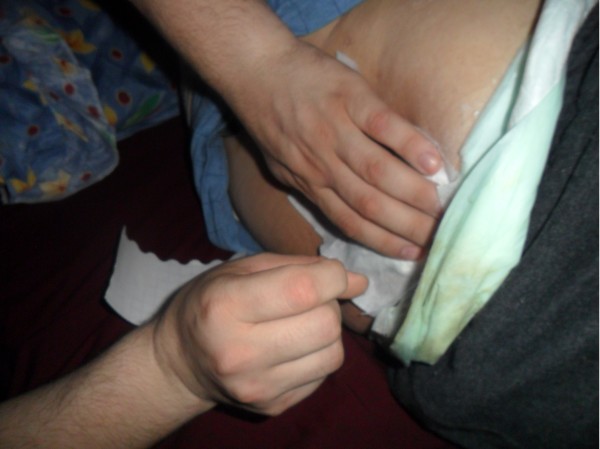
Softening of the black pine gum in a pot including hot water on the oven.

**Figure 7:**
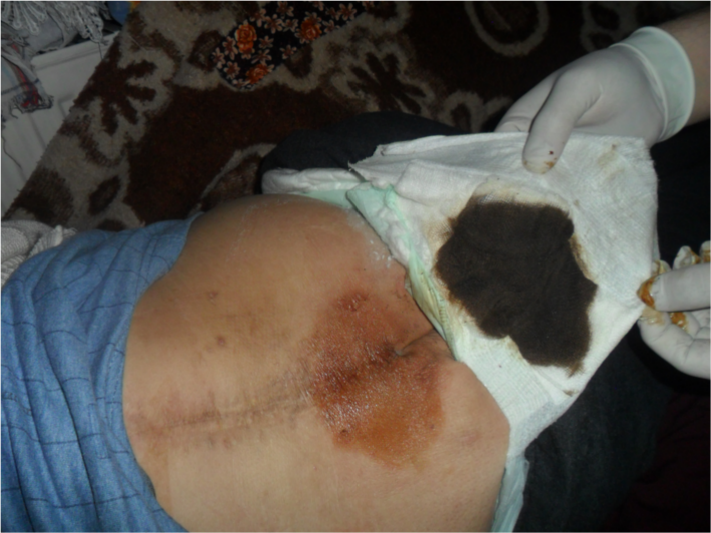
Preparing tar of the black pine and softened gum mixture to apply.

Tar has also been traditionally used to cure osteomyelitis. One local patient suffering from chronic osteomyelitis had lumbar abscesses. The patient had undergone fourteen different operations at different hospitals. The muscles of this patient had been badly damaged and could not heal because of recurrent bacterial infections. The patient was unable to move. The patient’s family decided to apply traditionally prepared tar to the abscesses in order to give the patient some relief (Figures 3 and 4). Fifty g of black pine gum was rolled into a sterile cloth and heated in a container in order to soften it (Figures 5 and 6). Fifty mL of tar was combined with 50 g of black pine gum and placed on the wounds (Figure 7). The wounds were covered with a piece of cloth for 24 hours. There was some skin irritation with this treatment. This process was repeated 10 times, with 3 day breaks between each session. After the second session, the pus was gone from the wounds. Before the sessions, the patient was unable to rotate to the right of left. However, after the sessions the patient was able to walk and be more comfortable. This method has been used for other skin wounds (Figures 4 and 7). Tar is also used to cure ulcers. For ulcers, water which contains 5–10 drops of tar is drunk before meals and after 5 sessions, the treatment is finished. Tar has been used for skin diseases such as eczema, acne, alopecia and fungus by the local people (Table 1).

Tar is used on animals as a cure for foot-and-mouth disease and viral infections. It is applied to the mouth sores of sheep and goats by local people. Tar is also used as a treatment for internal and external parasites. Villagers also use it to remove flies and ticks from sheep and goats. Local people make a mixture of tar and rock salt that is placed next to animals’ water pans and consumed by the animals to protect the digestive system from endoparasites. It has been discovered that after goats and sheep are sheered, mange can be prevented by rolling tar along the animals’ spinal cords. Additionally, the smell of tar acts as a snake repellant. Tar is also used to cure wounds inflicted by wolves. A mixture of 100 g barley flour and 25 mL tar is rolled onto wounds on animals. After 18 days, the wounds are healed (Table 1).

Plant species have been used as medicine, food, fodder, dye, firewood, construction materials and for other miscellaneous purposes in the districts of Sinanpaşa, Hocalar, and Dazkırı in Afyonkarahisar [9]. Kargıoğlu *et al.*[9] noted that a large part of Ahırdağı was covered by *Pinus nigra* Arn. subsp. *nigra* var. *caramanica* (Loudon) Rehder. Traditional medicinal plants have been used throughout Turkey to treat respiratory tract diseases, gastrointestinal diseases, kidney problems, diabetes, high cholesterol, rheumatic diseases, various cancer and cardiovascular problems and have also been used as fuel [3-5,7,8,13]. However, there are very few studies on tar. Tar is obtained from *Cedrus libani* A. Rich on the Taurus Mountains in Southern Turkey using traditional methods [16] and extracted from the wood of *Cedrus libani* using laboratory methods [23]. The traditional method for extracting tar from *Cedrus libani* is similar to the modern method. Furthermore, tar has been used to treat skin diseases such as eczema, acne, alopecia, fungus, mange and psoriasis for a long time in Anatolia, as well as to relieve inflammation and swelling [24,25]. Also, *Cedrus libani* tar has been used as a treatment for colds by applying tar to the back and chest, where it acts as a diaphoretic, skin softener, pain reliever and muscle relaxant [26], *Cedrus libani* tar is used as an antiseptic on dermatologic lesions and as a topical antifungal on the skin. *Juniperus drupacea* tar is used for respiratory tract and urinary tract diseases in addition to dermatological lesions [27]. *Pinus brutia* tar is used as an antiseptic in both the respiratory and urinary tracts and it is used externally for dermatological diseases [6]. The tar obtained from *P. nigra* and *P. brutia* is used as an oral antiseptic and for covering wounds [28]. Heated black tar is used to treat stomachaches in children. Tar is used to treat cracked hands and feet during the winter and for calluses [29]. Tar is also used to treat diseases such as smallpox, ulcers, diarrhea and pox [30]. Tar obtained from *Cedrus libani* is used to treat ulcers [16,31]. Tar is also given to people who were bitten by snakes after the poison has been leached out of the body [32] (Table 1).

Tar is also commonly used for additional diseases that were not covered by this study. For example, *Juniper sp.* tar is used as a cure for fluke disease, which is caused by the trematode parasite *Fasciola hepatica* that is pathogenic in sheep. It is applied to the palate of the animal [33]. Tar can be dried into a pellet that is used to treat internal parasites [29]. Tar smoke is used to kill the *Varroa destructor* parasite [34]. Additionally, tar is used as a repellant for the hive moth [6]. Pine tar is also used by local people for animals. If an animal overeats to the point where it may die, 5 or 6 g of tar is introduced into the animal’s mouth with a syringe and the animal is made to drink lots of water. The bacteria *Mycoplasma agalactiae* causes a reduction in goat’s milk. Tar is rolled into the feet of the animal infected with *Mycoplasma agalactiae*. After 3 days, their milk levels normalize. These findings are also described by Uysal [32] and Saday [26] (Table 1).

The fly *Tabanus bovinus* feeds on the blood of cows and is bothersome to the animal. Tar can be used externally on the wounds, scars and purple spots created by this fly. Local people stated that they used to use tar on their roofs when they were made of wood in order to prevent water leaks. When tar is used on houses, the smell also acts as a snake and rodent repellant. Similarly, Chavasse and Yap [35] reported that tar is used as a tick repellant by applying it to the skin of pets and animals and onto the skin or clothing of people (Table 1).

## Conclusion

The local people of Afyonkarahisar, in central Western Turkey have traditionally obtained tar from the Anatolian Black Pine [*Pinus nigra* Arn. subsp. *pallasiana* (Lamb.) Holmboe var. *pallasiana*] and have used the tar for a variety of purposes. The traditional knowledge of plants and their properties have usually been transferred from the elders to the youth. In this study, we interviewed local people to determine the traditional tar production method and its traditional usages. It is particularly interesting that tar is used to cure osteomyelitis by people who are generally above sixty and have a lot of practical experience with tar. More studies are need to determine the medicinal and veterinary usages of tar, as well as the mechanism of its healing properties.

## Consent

Written informed consent was obtained from the patient’s guardian/parent/next of kin for the publication of this report and any accompanying images.

## Competing interests

The authors declare that they have no competing interests.

## Authors’ contributions

SA main author, involved in the study design, conducting of interview, field work, literature Review and general data collection and systematization, wrote the first draft and MT wrote ms, designed figures, references and participated in fieldwork. M Konuk concluded the final version of this manuscript. MK is the main coordinator-supervisor of SA, project coordinator, diagnosed the plants, and participated in fieldwork. All authors read and approved the final manuscript.

## Authors’ information

SA is a doctoral student, MK is associate professor and MT is assistant professor at the Kocatepe University, corresponding author M. Konuk is professor at üsküdar University.
